# Biodistribution of Tc-99m Labeled Isoniazid Solid Lipid Nanoparticles in Wistar Rats

**Published:** 2018

**Authors:** Fereshteh Ghazizadeh, Solmaz Ghaffari, Seyedeh Fatemeh Mirshojaei, Mohammad Mazidid, Shirzad Azarmi

**Affiliations:** a *Department of Medical Nanotechnology, Faculty of Advanced Technologies, Pharmaceutical Sciences Branch, Islamic Azad University (IAUPS), Tehran, Iran. *; b *Young Researchers and Elite Club, Pharmaceutical Sciences Branch, Islamic Azad University (IAUPS), Tehran, Iran. *; c *Pharmaceutical Sciences Research Center, Pharmaceutical Sciences Branch, Islamic Azad University (IAUPS), Tehran, Iran. *; d *Radiation Application Research School, Nuclear Science and Technology Research Institute, AEOI, Tehran, Iran. *; e *Department of Pharmaceutics, Faculty of Pharmacy and Pharmaceutical Sciences, University of Alberta, Edmonton, Alta, Canada.*; f *Research Center for Pharmaceutical Nanotechnology, Faculty of Pharmacy, Tabriz University of Medical Sciences, Tabriz, Iran.*

**Keywords:** Isoniazid, Solid Lipid Nanoparticles, lyophilization, Tc-99m, Biodistribution, Gamma scintigraphy

## Abstract

In this study Isoniazid (INH) as one of the first line drugs in treatment of Tuberculosis was investigated to be loaded in Solid Lipid Nanoparticles (SLNs) for reducing hepatotoxicity as well as prolonging drug release. High shear homogenization method was performed to prepare INH SLNs. To compare biodistribution of INH before and after loading in SLNs, INH was labeled by Technetium 99 (Tc99) after derivatization. The particle size of the prepared SLNs was 167 and 200 nm before and after lyophilization, respectively. Loading efficiency was calculated using the reverse method and release study was performed by using the dialysis sack method. Loading efficiency was 98%, and more than 85% of the loaded drug released in 3 h. Differential Scanning calorimeter (DSC) studies were performed for evaluating of the probability of happening hydrogen bonds or other chemical interactions between cholesterol as carrier and isoniazid as active pharmaceutical ingredient. The results could support the probability of hydrogen bond formation between cholesterol and INH. Gamma Scintigraphy studies showed that after administering INH SLNs, longer drug retention in the body was obtained compared to free INH. Quantitative gamma counting showed that the concentration of INH in the liver and intestines could be decreased by using nanotechnology.

## Introduction

Isoniazid (INH) is an antibiotic used as a first-line agent for the prevention and treatment of both latent and active tuberculosis (TB). INH enters the mycobacterial cell by passive diffusion (1). INH itself is not toxic to the bacterial cell, but acts as a prodrug and is activated by the mycobacterial enzyme KatG (2, 3). Some mechanisms which cause to change mycobacterium resistance to INH were reported (4) as well as some side-effects including: hepatotoxicity and nephrotoxicity when used in combination with other drugs in treatment of TB and pulmonary fibrosis (5-7). To overcome drug resistance and adverse effects of TB chemotherapy agents and to improve patient compliance, novel drug delivery systems (NDDS) were introduced. Utilization of microparticles and nanoparticles including: nanocapsules, niosomes, liposomes as well as polymeric micelles were reported previously. One of the main goals in designing NDDS was preparing sustained drug delivery systems for pulmonary delivery or other routs of administration (8-10). Another problem in the treatment of TB is low bioavailibilty of rifampin when administered by INH. To resolve this problem other NDDS using crosslinked polymeric technology was designed by Toit *et al.* (11).

Gajendiran *et al.* studied INH polymeric nanoparticles to prolong drug release and enhance bioavailability (12). Some studies were carried out on targeting INH to intracellular mycobacterium using poly (lactide-co-glycolide) (PLGA) nanoparticles (13). Solid lipid nanoparticles (SLNs) are one of the nano size carries in drug delivery having many advantages like: biocompatibility, potential for sustaining drug release, simple preparation method, and low production cost. We chose SLNs to prepare a new drug delivery system of INH. The nano range size lets us design both oral and IV formulations. The purpose of this study was to prepare INH SLNs with proper drug loading and particle size as well as prolonged release profile. Biodistribution of INH-SLN was studied after IV and oral administration in comparison with free INH using radiolabeled INH.

## Experimental

*Materials*

Tween 80, ethanol, and acetone were purchased from Merck, Germany and were used as surfactant and oily phase solvents, respectively. Isoniazid as active pharmaceutical ingredients was prepared by Daroupakhsh Pharmaceutical Co, Iran. Dialysis membrane was purchased from Sigma, Germany. All ingredients which were used to prepare buffer phosphate solutions were bought from Merck, Germany. ^99m^Tc pertechnetate was supplied by AEOI, as ^99^Mo/^99m^Tc generator. All radioactivity measurements were carried out using Na (Tl) scintillation counter (ORTEC Model 4001M Minibin and Power Supply). 

*Methods*

*Derivatization of Isoniazid*

Since isoniazid does not have the required hetero atoms to make complex with Tc-99m, or the complex is not stable enough, we used it in a derivatized form. The derivatization of isoniazid was performed exactly according to Singh *et al.* procedure (14). briefly, 2 mg of INH was dissolved in 1 mL of distilled water and 4 mg of 2-Iminothiolane was dissolved in 2 mL of 1M triethanolamine being allowed to react for 1 h at 25-35 °C to introduce exogenous sulfydryl group to INH. 1.1 mg of sodium carbonate was added to the mixture with subsequent addition of chloroform, which is 1.5 times the volume of derivatized INH. The derivatized INH was washed with chloroform 2-3 times while vigorously shaking. The generation of SH group was confirmed by IR spectra.

*Radiocomplexation of derivatized Isoniazid*

Ten µg stannous chloride as reducing agent dissolved in HCl 0.1N was added to the derivatized isoniazid. The contents were mixed with 185-370 MBq of freshly eluted Tc-99m in physiological saline (2 mL) and incubated at room temperature for 20 min to achieve optimal labeling.

The labeling yield and radiochemical purity were determined by Thin Layer Chromatography (TLC). The reaction product was spotted on silica gel ITLC-SA strips (Gelman Sciences Inc., USA), 10 × 1.5 cm^2^ sheets, and developed in THF as the mobile phase. After developing, they were scanned by TLC Scanner (GINA-Star TLC-raytest Isotopenmeßgeräte GmbH). In this system, free pertechnetate was moved away with solvent front leaving ^99m^Tc in RF = 0.9 and the complex was seen in the RF = 0.5-0.7 and reduced hydrolyzed technetium at the point of application.

*Preparation of Isoniazid SLNs*

Isoniazid SLNs were prepared using the method which was presented by Varshosaz *et al.* 2010 (15). In brief, 750 mg cholesterol was dissolved in 24 mL mixture of ethanol and acetone in 3 to 1 ratio by heating at 50-60 °C. Five-hundred mg of free isoniazid and/or Tc-99m linked isoniazid dissolved in 120 mL deionized water containing 1% tween 80 for *in-vitro* and *in-vivo* studies, respectively. The hot oily phase was added to water phase and mixture was homogenized at 11000 rpm for 7 min. SLNs formed during cooling of mixture to room temperature. 

*Freeze drying procedure*

To increase stability of prepared nanoparticles and inhibit aggregation of particles during shelf life particles were freeze-dried using 5% mannitol as cryoprotectant at -40 °C as pre-freezing temperature for 24 h. Lyophilization was carried out using freeze-dryer (Lyotrap/Plus, UK) at -40 °C and the pressure was 0.4 bar (16).

*In-vitro evaluation of Isoniazid SLNs*

*Determination of loading efficiency*

For determination of drug loading efficacy (LE%) of INH solid lipid nanoparticles, the samples were centrifuged at 25,000 rpm for 40 min at -4 °C using Sigma Laboratories Ltd. centrifuge (Germany). The drug concentration in the supernatant was analyzed using UV spectrophotometer (Shimadzu, Japan) at 263 nm and LE% was calculated using reverse method.

*Particle size analysis*

The evaluation of particle size as well as zeta potential was done using Malvern zeta sizer (ZEN3600) before and after freeze drying.

*Morphology studies*

Morphology of the nanoparticles was investigated by scanning electron microscopy (SEM). The nanoparticles were mounted on aluminum stubs; sputter- coated with a thin layer of Au/Pd and examined using an SEM (SEM XL30, Philips, Netherlands) before and after freeze drying.

*Drug release testing*

Release study was performed using dialysis sack method by DO405 dialysis membrane (Sigma, Germany). Five mL of formulation was placed in dialysis membrane and immersed in 50 mL phosphate buffer solution pH 7.4. Two mL samples were withdrawn in predetermined time intervals and drug concentration was measured at 263 nm using UV spectrophotometer (Shimadzu, Japan). Dilution of withdrawn samples was done if needed. 

*Differential scanning calorimeter (DSC) studies*

For evaluation of probability of happening hydrogen bonds or other chemical interactions between cholesterol as carrier and isoniazid as active pharmaceutical ingredient, DSC studies were done. This investigation could confirm the results of release profile as well.

*In-vivo studies*

To investigate biodistribution of INH, 180-200 g male Wistar rats from animal house of the School of Pharmacy and Pharmaceutical Sciences, Tehran University of Medical Sciences were used for the *in-vivo* studies. The animals were housed in colony cages with free access to standard chow pellets and water, under controlled conditions (22 ± 2 °C, 12 h light-dark cycle, 55–65% humidity), and placed in the laboratory for 3-4 days during the acclimatization period.

The animal study was approved by the guidelines of the ethical committee of Islamic Azad University of Medical Sciences. 

One-hundred µL of free INH and INH SLNs in equal concentration was injected to tail vein of rats, in other groups the same doses were given to rats orally. Each animal received 100 µL of IV ketamine solution (10%) before drug administration. Then 20 and 75 min after IV and oral administration respectively, the biodistribution of INH was investigated using gamma scintigraphy analysis and the results were confirmed by removing lung, heart, liver, kidneys, spleen, stomach as well as blood and calculating %ID/g of each organ. The rats in each subgroup were killed using CO2 gas in the desired time point to detect drug concentration in desired organs.

*Gamma Scintigraphy Analysis*

*In-vivo* evaluation of pharmaceutical products was achieved by γ-scintigraphicimaging of the particles deposited in the bodies of animals after oral and IV administration. For all studies a single headed camera (PICKER-PRISM1000, USA) employing a low energy gamma high resolution collimator was used.

*In-vivo* *quantitative studies of isoniazid SLNs after oral and IV administration*

Quantitative gamma counting was performed on ORTEC model 4001M γ-system wellcounter. %ID/g of each organ was calculated using the following equation in which ID is injected dose and pure M was pure weight of each organ(s) (17, 18).

%ID/Organ Weight = Each Count/Total Count × 100/Pure M

## Results and Discussion

*Particle size analysis*

Particle size distribution was evaluated by Dynamic light scattering (DLS) technique. The results show that particle size is 167 nm with polydispersity index (Pdi) equal to 0.25. Size enlargement after freeze drying was not significant and also no aggregation was observed due to using cryoprotectant in freeze drying step.

*Morphology Studies*

**Figure 1:**
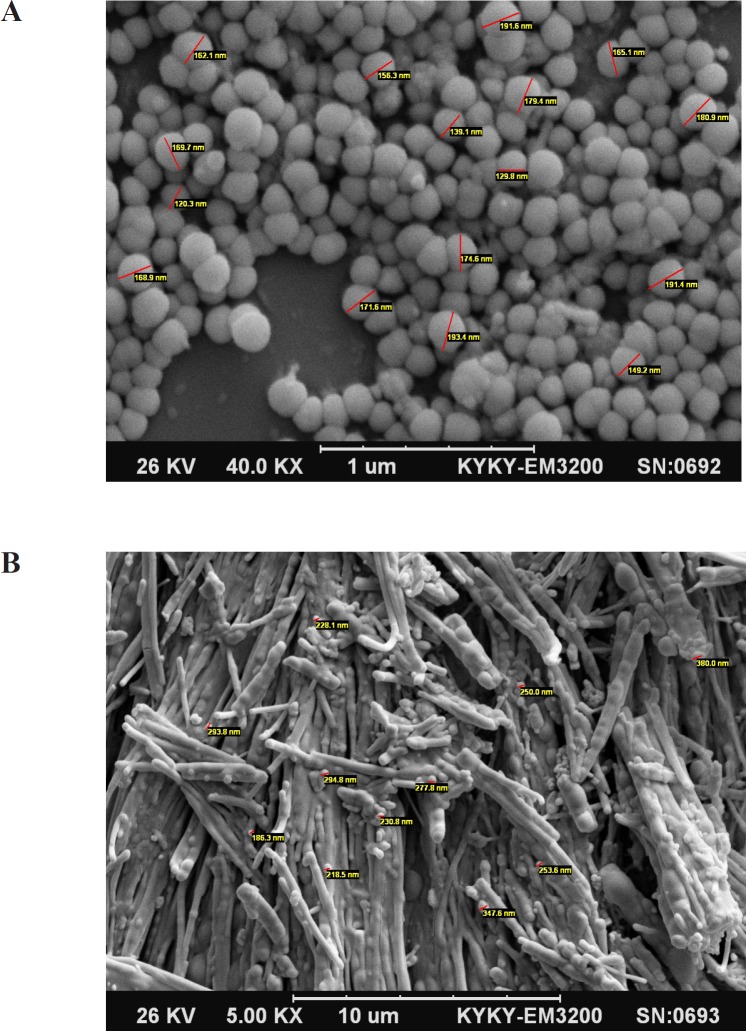
(A) Scanning electron microscopy picture of INH solid lipid nanoparticles before freeze drying. (B) Scanning electron microscopy picture of INH solid lipid nanoparticles after freeze drying

SEM photographs showed that the desired SLNs have spherical shape and the particle size was confirmed by this technique in comparison with DLS. Figures 1A and 1B. Show SEM pictures of INH loaded SLNs before and after freeze drying. The spherical shapes of particles were remained after freeze drying.

*Loading efficiency and Release study*

**Figure 2:**
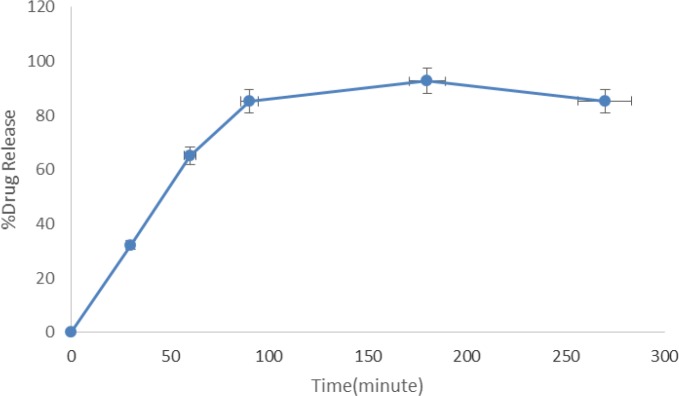
Release profile of INH solid lipidnanoparticles

Loading efficiency (%LE) was 98% for INH. Release studies show that more than 90% of loaded INH was released after 3 h, no significant burst effect was observed in the first hour before and after freeze drying. Figure 2 shows the drug release profile of INH from INH-SLNs.

*DSC Studies*

**Figure 3:**
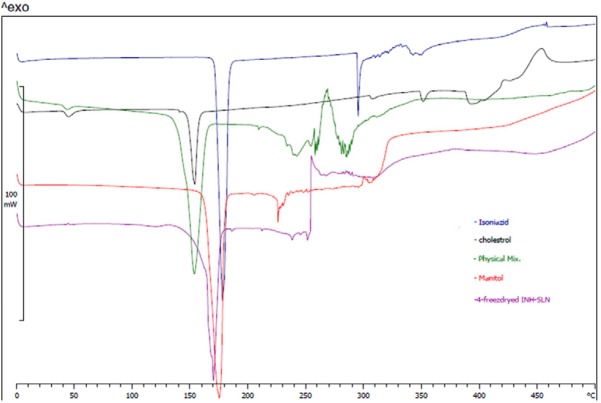
DSC thermograms of raw materials and freeze-dried SLNs of INH

DSC thermograms showed shifting of cholesterol and INH melting points from 155 and 180 °C to 170 °C in INH-SLNs. Chemical structure of INH and cholesterol could support the probability of hydrogen bond formation between cholesterol and INH which resulted in release profile as well. Figure 3 shows DSC thermograms.

*Derivatization of INH*

**Figure 4:**
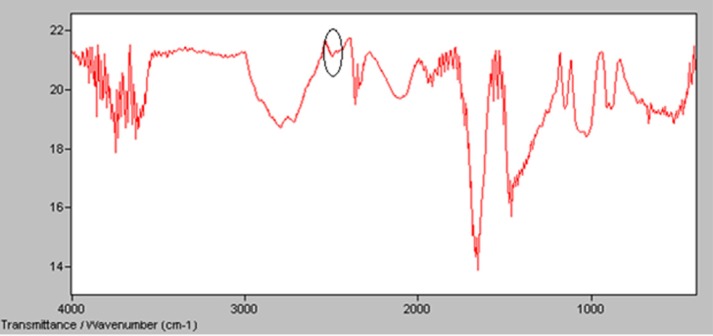
IR spectra of derivatized INH

Derivatization of INH using 2-iminothiolane was evaluated by IR spectra in which the peak of SH group was established at 2450 cmˉ^1 ^(14). Figure 4 shows the presence of significant peak in 2450 cm^-1^ which could confirm formation of SH group that was needed for radiolabeling of INH (14).

*Purity of INH-Tc99 complex*

**Figure 5:**
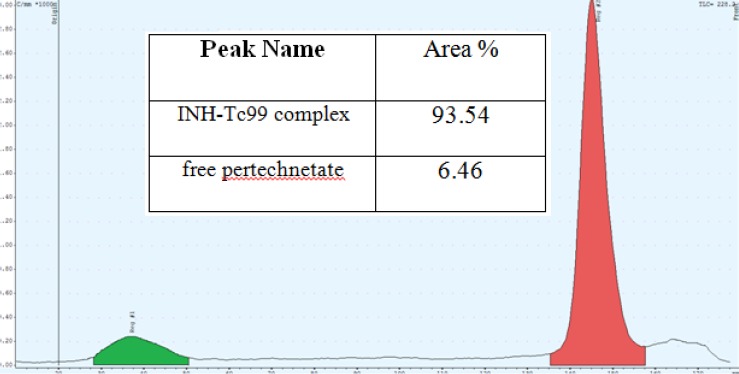
Thin Layer Chromatography result, INH-Tc99 complex purity

The results showed that total amount of free pertechnetate and hydrolyzed Tc was less than 7%. On the other hand, purity of INH-Tc99 was equal to 93.54% which is in acceptable level. Figure 5 shows the thin layer chromatography result.

*In-vivo evaluation of isoniazid SLNs*

*Gamma Scintigraphy studies*

**Figure 6:**
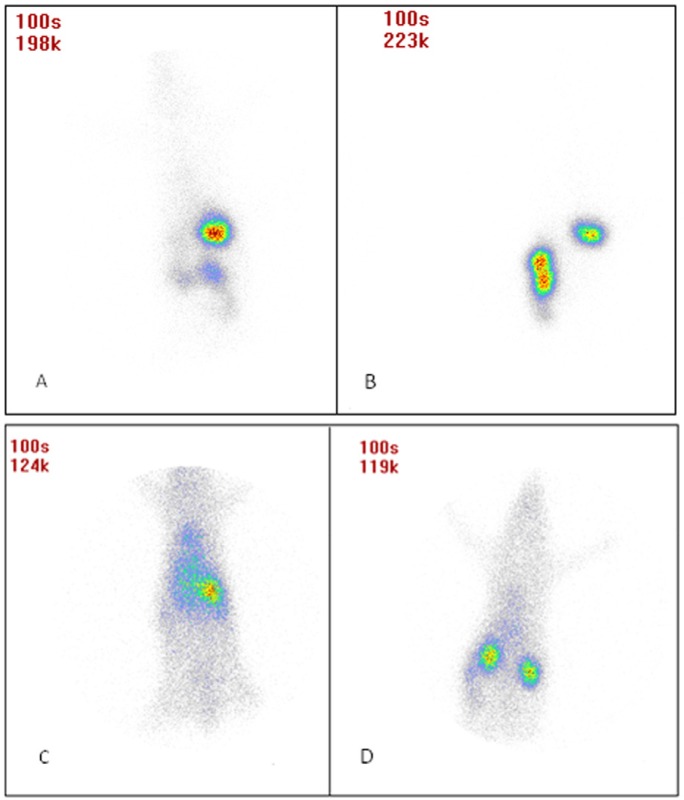
Gamma scintigraphy photographs of rats receiving (A) oral free INH after 75 min, (B) oral INH SLNs after 75 min, (C) iv free INH after 20 min, and (D) iv INH SLNs after 20 min

Figures 6A-D shows animal photos after dosing. Results show that after administering INH SLNs, longer drug retention was obtained in the body compared to free INH. Also after oral administration, free INH was removed from stomach faster than INH SLNs. The remaining of INH in the blood is significantly much more than free INH after IV administration, and the same results were seen after oral administration but not as significant as IV dosing. 

*Quantitative biodistribution studies*

%ID/Organ weight was calculated for each desired organ and Table 1 shows results in this regard.

**Table 1:** %ID/ organ weight

**Organ name**	**%ID/g after IV injection, Free INH**	**%ID/g after IV injection, INH-SLNs**	**%ID/g after oral administration, Free INH**	**%ID/g after oral administration, INH-SLNs**
Lung	0.54 ± 0.04	0.65 ± 0.05	0.2 ± 0.03	0.54 ± 0.02
Liver	4.59 ± 0.43	0.8 ± 0.03	0.03 ± 0.005	0.33 ± 0.01
Kidneys	4.93 ± 0.35	1.86 ± 0.07	0.08 ± 0.006	0.43 ± 0.03
Stomach	0.10 ± 0.01	2.72 ± 0.2	11.36 ± 1.02	13.48 ± 1.03
Intestine	0.36 ± 0.02	0.40 ± 0.06	4.19 ± 0.23	1.59 ± 0.4
Spleen	0.01 ± 0.002	0.32 ± 0.04	0.02 ± 0.001	0.17 ± 0.04
Heart	0.41 ± 0.02	0.4 ± 0.01	0.02 ± 0.002	0.19 ± 0.01
Blood	0 ± 0	1.22 ± 0.1	0.04 ± 0.003	0.55 ± 0.07
Rest of bodies	0.12 ± 0.01	0.19 ± 0.01	0.01 ± 0.001	0.09 ± 0.01

*IV administration results*

Based on the results of Table 1 the amount of INH in the liver and kidneys were decreased after SLN delivery in comparison with free INH administration after IV administration (*p*-value < 0.05)

IV administration of INH SLNs caused more INH detection in the lungs in comparison with free drug. Also more amount of INH was detected in stomach and spleen after IV administration of INH SLNs (*p*-value < 0.05) and the same results were observed in the intestines but the organ results were not as significant as other organs. 

*Oral administration results*

After oral administration of INH SLNs significantly more amount of drug in all studied body organs were detected except spleen (*p*-value < 0.05).

Comparison between oral and IV administration of INH SLNs showed that more amount of drug in the lungs could be detected when INH SLNs were administered IV, but the rout of administration could not cause decreasing the amount of drug in liver and kidneys. 

Previously some studies were carried out to demonstrate that using nanotechnology could cause decreasing MIC and MBC of antibacterial agents (15-19). 

INH acts as an antibacterial agent after diffusing in bacteria cell. Since one of the mechanisms of SLNs is helping to facilitate diffusion of anti-bacterial drugs in to bacteria cell using different mechanisms. Probably SLNs could decrease the effective dose of INH by decreasing MIC and MBC as well as many other antibiotics. Also, other study was carried out to increase release duration of INH using SLN drug delivery system (20). In that study scientist could improve bioavailability of INH as well. They administered the designed SLNs just orally to evaluate plasma concentration of INH SLNs in comparison with free INH.

In brief it seems that using SLNs could lead to increase the amount of INH in the lungs as target organ in tuberculosis treatment. So, probably with less drug dosing, the lungs infections could be controlled. Most of the times less drug dosing help us having less drug side effects as well. Of course more studies should be carried out to confirm the results of the presented study. 
